# Proteomic profiling of liver tissue from the *mdx*-*4cv* mouse model of Duchenne muscular dystrophy

**DOI:** 10.1186/s12014-018-9212-2

**Published:** 2018-10-29

**Authors:** Sandra Murphy, Margit Zweyer, Michael Henry, Paula Meleady, Rustam R. Mundegar, Dieter Swandulla, Kay Ohlendieck

**Affiliations:** 1 Department of Biology, Maynooth University, National University of Ireland, Maynooth, Co. Kildare, Ireland; 2 Institute of Physiology II, University of Bonn, 53115 Bonn, Germany; 3 National Institute for Cellular Biotechnology, Dublin City University, Dublin 9, Ireland

**Keywords:** Dystrophin, Dystrophinopathy, FABP5, Fatty acid binding protein, Fatty acid metabolism, Ferritin, Neuromuscular disease

## Abstract

**Background:**

Duchenne muscular dystrophy is a highly complex multi-system disease caused by primary abnormalities in the membrane cytoskeletal protein dystrophin. Besides progressive skeletal muscle degeneration, this neuromuscular disorder is also associated with pathophysiological perturbations in many other organs including the liver. To determine potential proteome-wide alterations in liver tissue, we have used a comparative and mass spectrometry-based approach to study the dystrophic *mdx*-*4cv* mouse model of dystrophinopathy.

**Methods:**

The comparative proteomic profiling of *mdx*-*4cv* versus wild type liver extracts was carried out with an Orbitrap Fusion Tribrid mass spectrometer. The distribution of identified liver proteins within protein families and potential protein interaction patterns were analysed by systems bioinformatics. Key findings on fatty acid binding proteins were confirmed by immunoblot analysis and immunofluorescence microscopy.

**Results:**

The proteomic analysis revealed changes in a variety of protein families, affecting especially fatty acid, carbohydrate and amino acid metabolism, biotransformation, the cellular stress response and ion handling in the *mdx*-*4cv* liver. Drastically increased protein species were identified as fatty acid binding protein FABP5, ferritin and calumenin. Decreased liver proteins included phosphoglycerate kinase, apolipoprotein and perilipin. The drastic change in FABP5 was independently verified by immunoblotting and immunofluorescence microscopy.

**Conclusions:**

The proteomic results presented here indicate that the intricate and multifaceted pathogenesis of the *mdx*-*4cv* model of dystrophinopathy is associated with secondary alterations in the liver affecting especially fatty acid transportation. Since FABP5 levels were also shown to be elevated in serum from dystrophic mice, this protein might be a useful indicator for monitoring liver changes in X-linked muscular dystrophy.

## Background

The X-linked recessively inherited disorder Duchenne muscular dystrophy is primarily a monogenetic disease of the skeletal musculature [1]. The almost complete loss of the membrane cytoskeletal protein dystrophin triggers a complex pathogenesis in voluntary muscles that is characterized by highly progressive fibre degeneration, abnormal calcium homeostasis, fatty tissue replacement, reactive myofibrosis and sterile inflammation [2–4]. However, besides skeletal muscle wasting, patients afflicted with dystrophinopathy also exhibit a multitude of secondary alterations in the body, including respiratory insufficiency, cardiomyopathy, renal and urinary tract dysfunction, impaired bone metabolism, growth retardation, contractures of tendons, scoliosis and gastrointestinal symptoms [5–8], as well as non-progressive cognitive deficiencies in a subset of patients [9]. In addition, the pathological survey of general organ changes in Duchenne patients revealed in most cases a marked atrophy of the liver, whereby liver shrinkage increased with patient age [10].

Liver atrophy was shown to occur concurrently with loss of skeletal muscle mass [10, 11] and liver changes were reported to be linked to heart failure in older Duchenne patients [12]. Muscular dystrophy was also shown to be associated with an increased susceptibility to drug-induced hepatotoxicity [13, 14]. An interesting connection between dystrophic skeletal muscles and abnormal liver function is related to altered levels of the amino acid taurine. Physiological levels of taurine are crucial for the normal functioning of skeletal muscles and are related to the facilitation of excitation–contraction coupling, the modification of intracellular calcium homeostasis, membrane stabilization, the support of anti-oxidant defence mechanisms and the osmo-regulation of cellular volume [15]. Deficiency of taurine in dystrophic *mdx* mice was shown to be associated with perturbations in taurine transport and altered metabolism in the liver [16]. Thus, the dystrophic phenotype does not only feature impaired taurine metabolism in the skeletal musculature, but is also characterized by pathophysiological alterations of taurine handling in the liver, which can be rectified by increasing dietary taurine causing improved contractile strength [17]. This positive effect of taurine is probably mediated via anti-inflammatory, anti-oxidant and cyto-protective mechanisms [18]. Another interesting liver-related abnormality in the *mdx* mouse model of dystrophinopathy is decreased liver glycogen levels and hyperglycemia [19], which agrees with a mild glucose intolerance observed in Duchenne patients [20, 21]. Based on these findings, it was of interest to investigate whether the dystrophic phenotype is associated with proteome-wide changes in liver tissue.

Mass spectrometry-based proteomics has previously been used to study a variety of tissues and organs from dystrophic animal models, as summarized in several recent review articles [22–24]. The proteomic profiling of the *mdx*-*4cv* mouse model of Duchenne muscular dystrophy, which is characterized by a low frequency of revertant fibres [25–27], has established considerable changes in the dystrophin-deficient skeletal musculature, heart, brain and serum. Distinct alterations were shown to occur in proteins involved in the maintenance of the cytoskeletal network, the basal lamina and associated extracellular matrix, the regulation of ion homeostasis and excitation–contraction coupling, energy metabolism and the cellular stress response in various tissues from dystrophic animals [28–34]. In analogy, the comparative screening of crude liver extracts from *mdx*-*4cv* mice outlined in this report has shown distinct proteome-wide changes in a variety of enzymes, metabolite transporters, ion binding proteins, signalling molecules and structural proteins. Muscular dystrophy-related perturbations were identified to affect especially metabolic pathways in the liver, whereby the most drastically increased protein was identified as the FABP5 isoform of fatty acid binding protein and the most decreased protein as phosphoglycerate kinase isoform PGK1.

## Methods

### Materials

For the comparative proteomic analysis of liver tissue from wild type versus dystrophic *mdx*-*4cv* mice, a variety of general analytical grade reagents and materials were obtained from GE Healthcare (Little Chalfont, Buckinghamshire, UK), Bio-Rad Laboratories (Hemel-Hempstead, Hertfordshire, UK) and Sigma Chemical Company (Dorset, UK). National Diagnostics (Atlanta, GA, USA) supplied ultrapure acrylamide stock solutions. Sequencing grade-modified trypsin, Lys-C and Protease Max Surfactant Trypsin Enhancer were purchased from Promega (Madison, WI, USA) and Whatman nitrocellulose transfer membranes came from Invitrogen (Carlsbad, CA, USA). The chemiluminescence substrate and protease inhibitors were obtained from Roche Diagnostics (Mannheim, Germany). Primary antibodies were from Abcam, Cambridge, UK (antibodies ab129203 to fatty acid binding protein FABP-1; ab69090 to ferritin light chain; ab14734 to the voltage-dependent anion channel VDAC-1; ab85366 to carbonic anhydrase isoform CA3; and ab2413 to fibronectin), Novus Biologicals, Littleton, CO, USA (NBP2-15492 to asporin; and NBP1-80671 to the mitochondrial outer membrane protein translocation pore subunit TOM22), Leica Biosystems, Wetzlar, Germany (antibody NCL-DYS1 [clone Dy8/6C5] to dystrophin) and Cell Signaling Technology, Leiden, Netherlands (antibody 39926T to fatty acid binding protein FABP5). Chemicon International (Temecula, CA, USA) provided peroxidase-conjugated secondary antibodies. For immunofluorescence microscopy, normal goat serum, goat anti-rabbit Alexa Fluor 488 and goat anti-mouse IgG RRX (Rhodamine Red-X) were purchased from Molecular Probes, Life Technologies (Darmstadt, Germany) and Jackson ImmunoResearch (West Grove, PA, USA), respectively. The embedding medium Fluoromount G was from Southern Biotech (Birmingham, AL, USA).

### Dystrophic *mdx*-*4cv* mouse model of Duchenne muscular dystrophy

The *mdx*-*4cv* mouse model is one of four chemical variants of the *mdx* mouse [27], generated on the C57/BL6 background by chemical mutagenesis using N-ethyl-nitrosourea [35]. The resulting mutation at base 7916 in exon 53 of the dystrophin gene introduces a premature stop codon, thus abrogating dystrophin synthesis. For the identification of global alterations in the liver proteome of dystrophic mice, whole liver samples from 6-month-old control C57/BL6 mice and age-matched *mdx*-*4cv* mice were obtained from the Bioresource Unit of the University of Bonn [29]. Mice were kept under standard conditions and all procedures adhered to German legislation on the use of animals in experimental research. The animals were sacrificed by cervical dislocation and livers were carefully dissected and quick-frozen in liquid nitrogen. Samples were transported to Maynooth University in accordance with the Department of Agriculture (animal by-product register number 2016/16 to the Department of Biology, National University of Ireland, Maynooth) on dry ice and stored at − 80 °C prior to analysis.

### Preparation of liver homogenates from wild type and dystrophic mice

For the preparation of crude extracts, 100 mg of liver tissue from 6-month-old *mdx*-4cv mice (n = 4) and age-matched wild type mice (n = 4) was finely chopped and homogenised in 10 volumes of lysis buffer (6 M urea, 2 M thiourea, supplemented with a protease inhibitor cocktail [30]), using a hand-held IKA T10 Basic Homogeniser (IKA Labortechnik, Staufen, Germany). Homogenates were kept on ice for 1.5 h and were subsequently centrifuged at 14,000×*g* for 20 min at 4 °C [36]. The protein-containing supernatant was removed and used for comparative proteomic analysis.

### Sample preparation for label-free liquid chromatography mass spectrometry

Liver homogenates were treated using the Ready Prep 2D clean up kit from BioRad Laboratories, and re-suspended in label-free solubilisation buffer (6 M urea, 2 M thiourea, 10 mM Tris, pH 8.0 in LC–MS grade water). The protein concentrations were determined using the Bradford assay system [37] and sample volumes were then equalised with label-free solubilisation buffer. For mass spectrometric analysis, 10 µg of liver homogenate was reduced with 10 mM dithiothreitol for 30 min at room temperature, and alkylated with 25 mM iodoacetamide in 50 mM ammonium bicarbonate for 20 min at room temperature in the dark. A further 10 mM dithiothreitol was added and samples were incubated for 15 min at room temperature in the dark, to quench any unreacted iodoacetamide [38]. Protein samples were first digested with sequencing grade Lys-C at a ratio of 1:100 (protease:protein) and incubated at 37 °C for 4 h. Samples were diluted with four times the initial sample volume with 50 mM ammonium bicarbonate, and 1 μl of a 1% w/v solution of Protease Max Surfactant Trypsin Enhancer was added [39], followed by digestion with sequencing grade-modified trypsin at a ratio of 1:25 (protease:protein) overnight at 37 °C. Proteolytic digestion was halted by the addition of 2% trifluoroacetic acid (TFA) in 20% acetonitrile (ACN) (3:1 (v/v) dilution). The peptides were purified using Pierce C18 spin columns from Thermo Fisher Scientific (Dublin, Ireland), dried through vacuum centrifugation and re-suspended in an appropriate volume of loading buffer (2% ACN, 0.05% TFA in LC–MS grade water). Peptide suspensions were vortexed, sonicated, and centrifuged briefly at 14,000×*g* before being transferred to mass spectrometry vials [31].

### Label-free liquid chromatography mass spectrometry

Reverse-phased capillary high pressure liquid chromatography was carried out using the UltiMate 3000 nano system (Thermo Scientific) coupled directly in-line with the Thermo Orbitrap Fusion Tribrid Mass Spectrometer (Thermo Scientific). The digested samples were loaded onto the trapping column (PepMap100, C18, 300 μm × 5 mm) at a flow rate of 25 μl/min with 2% (v/v) ACN, 0.1% (v/v) TFA for 3 min before being resolved onto an analytical column (Easy-Spray C18 75 μm × 500 mm, 2 μm bead diameter column). Peptides were eluted using the following binary gradient: solvent A (0.1% (v/v) formic acid in LC–MS grade water) and 2–27.5% solvent B (80% (v/v) ACN, 0.08% (v/v) formic acid in LC–MS grade water) for 110 min at a flow rate of 300 nl/min. For peptide ionization, a voltage of 1.9 kV was applied and a capillary temperature of 320 °C was used. Data-dependent acquisition with full scans in the 375–1500 m/z range was performed using an Orbitrap mass analyser with a resolution of 120,000 (at m/z 200), a targeted automatic gain control (AGC) value of 4E+05 and a maximum injection time of 50 ms. The number of selected precursor ions for fragmentation was determined by the top-speed acquisition algorithm. Selected precursor ions were isolated in the Quadrupole instrument with an isolation width of 1.6 Da. Peptides with a charge state of 2+ to 6+ were analysed and a dynamic exclusion was applied after 60 s. Precursor ions were fragmented using higher energy collision-induced dissociation (HCD) with a normalized collision energy of 28%, and resulting MS/MS ions were measured in the linear ion trap. The typical MS/MS scan conditions were as follows: a targeted AGC value of 2E+04 and a maximum fill time of 35 ms.

### Protein profiling by label-free LC–MS/MS

Progenesis QI for Proteomics software (version 3.1; Non-Linear Dynamics, a Waters company, Newcastle upon Tyne, UK) was used for the quantitative analysis of liver homogenates from wild type versus *mdx*-*4cv* mice. To account for potential drifts in retention time, a reference run was selected and all other runs were aligned to this run. The following settings were used to filter peptide features: (1) peptide features with ANOVA ≤ 0.05 between experimental groups, and (2) mass peaks with charge states from + 1 to + 5 and (3) greater than one isotope per peptide [31]. A Mascot generic file (mgf) was generated from all exported MS/MS spectra, which was used for peptide and protein identification via Proteome Discoverer 2.1 (Thermo Scientific) using Sequest HT against the UniProtKB-SwissProt *Mus musculus* database. The following search parameters were used: (1) peptide mass tolerance set to 10 ppm, (2) MS/MS mass tolerance set to 0.6 Da, (3) up to two missed cleavages, (4) carbamidomethylation set as a fixed modification and (v) methionine oxidation set as a variable modification. For re-importation back into Progenesis LC–MS software as a PepXML file only highly confident peptides with a FDR of ≤ 0.01 as determined by Percolator validation in Proteome Discoverer were allowed [40]. The following criteria were applied to assign proteins as positively identified: (1) an ANOVA score between experimental groups of ≤ 0.05, (2) proteins with ≥ 2 unique peptides matched and (3) a fold change ≥ 1.5. A heat map illustrating protein abundances for statistically significant and differentially abundant proteins was designed using Perseus software version 1.6.2.1. The normalised abundance values of differentially abundant proteins were determined using Progenesis QI for Proteomics and were loaded as a txt file into Perseus and the data was log2 transformed. Hierarchical clustering was then performed on *Z*-score normalised intensity values by clustering both samples and proteins using Euclidean distance and average linkage. The freely available software packages PANTHER [41] and STRING [42] were used to identify protein class and characterise potential protein interactions, respectively.

### Comparative immunoblot analysis

Comparative immunoblotting was used as an orthogonal method for the independent verification of interesting proteins with a differential expression pattern in the liver of the *mdx*-*4cv* mouse model of Duchenne muscular dystrophy. Liver homogenates for immunoblot analysis were prepared in 2 × standard Laemmli-type buffer for one-dimensional sodium dodecyl sulfate polyacrylamide gel electrophoresis (SDS-PAGE) [43], heated at 97 °C for 7 min and loaded onto hand-cast 10% SDS-PAGE gels. Proteins were transferred to nitrocellulose membranes, blocked in a milk protein solution (2.5% (w/v) fat-free milk powder in 10% phosphate-buffered saline) and incubated overnight in appropriately diluted primary antibody. Following a number of washing steps, membranes were incubated with peroxidase-conjugated secondary antibodies, and immuno-decorated protein bands visualized with the help of the enhanced chemiluminescence technique [33]. Densitometric scanning and statistical analysis of immunoblots was performed using a HP PSC-2355 scanner and ImageJ software (NIH, Bethesda, MD, USA) along with Graph-Pad Prism software (San Diego, CA, USA), in which statistical significance was based on a *p* value ≤ 0.05.

### Comparative histology and immunofluorescence microscopy

In order to establish the loss of the skeletal muscle dystrophin isoform Dp427-M in the *mdx*-*4cv* mouse model of dystrophinopathy and correlate it to potential changes in the liver, histological haematoxylin and eosin staining, as well as immunofluorescence microscopy were carried out by standardized methodology. Freshly dissected liver and muscle specimens from 6-month-old wild type and *mdx*-*4cv* mice were quick-frozen in liquid nitrogen-cooled isopentane and 10 µm sections cut in a cryostat [29]. For dystrophin immuno-staining, unfixed cryosections were boiled in phosphate-buffered saline for 5 min as previously described in detail [44]. For FABP5 staining, sections were fixed in a 1:1 (v/v) mixture of methanol and acetone for 10 min at room temperature and then blocked with 1:20 diluted normal goat serum for 30 min at room temperature. Primary antibodies to dystrophin and FABP5 were diluted appropriately in phosphate-buffered saline for overnight incubation at 4 °C. Tissue specimens were carefully washed and then incubated with fluorescently-labelled secondary antibodies, using either 1:200 diluted anti-rabbit Alexa Fluor 488 antibody or 1:200 diluted anti-mouse RRX antibody for 45 min at room temperature. Nuclei were counter-stained with 1 μg/ml bis-benzimide Hoechst 33342. Antibody-labelled liver and muscle tissue sections were embedded in Fluoromount G medium and viewed under a Zeiss Axioskop 2 epifluorescence microscope equipped with a digital Zeiss AxioCam HRc camera (Carl Zeiss Jena GmbH, Jena, Germany). For Sudan Black staining, fresh tissue sections were fixed in 10% formalin, washed under tap water, rinsed with distilled water and then exposed twice for 5 min to propylene glycol. Incubation with Sudan Black B (0.7 g dye in 100 ml propylene glycol) was carried out for 7 min with agitation, followed by 3 min in 85% propylene glycol, rinsing with distilled water, washing with tap water, another rinsing step with distilled water and finally mounting in an aqueous mounting media of glycerin jelly.

## Results

### Proteomic profiling of crude extracts from mouse liver

**Fig. 1:**
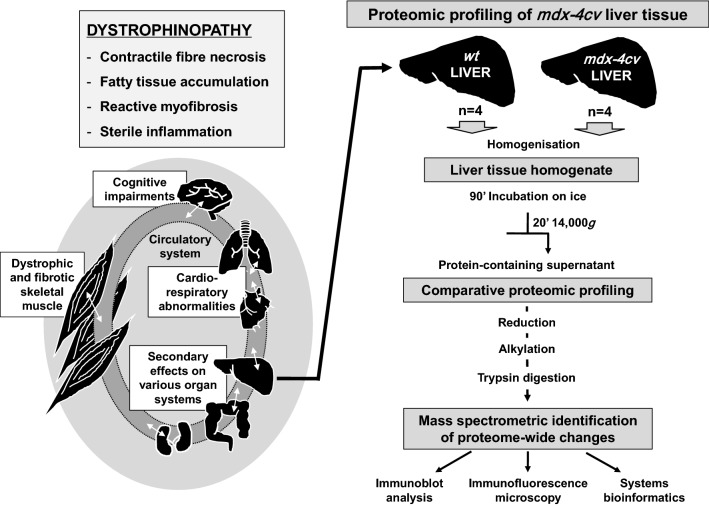
Overview of the complex multi-system pathophysiology of dystrophinopathy and proteomic workflow to analyse the *mdx*-*4cv* liver. Duchenne muscular dystrophy is caused by primary abnormalities in dystrophin and triggers progressive skeletal muscle wasting, cardio-respiratory abnormalities and cognitive impairments. In addition, X-linked muscular dystrophy is also characterized by secondary effects on a variety of organ systems including the liver. Proteome-wide changes in liver tissue were determined by comparative proteomics using the dystrophic *mdx*-*4cv* mouse model of Duchenne muscular dystrophy. Results obtained by mass spectrometry using an Orbitrap Fusion Tribrid apparatus were analysed by systems bioinformatics, and key findings were confirmed by verification studies employing immunoblotting and immunofluorescence microscopy

For the comparative proteomic profiling of liver tissue from wild type versus the dystrophic *mdx*-*4cv* model of Duchenne muscular dystrophy, total protein extracts were analysed by mass spectrometry. The pathophysiological complexity of dystrophinopathy and the bioanalytical approach used to evaluate proteome-wide changes in liver tissue is outlined in the flow chart of Fig. 1. In the wild type, the multi consensus analysis recognized a total of 2536 protein species, which were identified from a total of 18,283 peptides and 16,976 unique peptides. Of the total number of liver-associated proteomic hits from wild type mouse, 1607 proteins were identified by a minimum of two unique peptides. The analysis of the multi consensus data of the dystrophic *mdx*-*4cv* model recognized a total of 2463 proteins, which were identified from a total of 18,543 total peptides and 17,069 unique peptides. Of the total number of liver-associated proteomic hits from the *mdx*-*4cv* mouse model, 1618 proteins were identified by a minimum of two unique peptides.

Since the below outlined proteomic findings suggest considerable perturbations of fatty acid transportation and lipid metabolism, it was of interest to confirm the presence of key transporters and enzymes of lipid metabolism in this proteomic screening study. Important metabolic proteins were identified with high confidence in normal liver tissue including markers of (1) fatty acid transportation, including the main liver-specific fatty acid binding protein isoform FABP1 (L-FABP; P12710), as well as the intestinal isoform FABP2 (I-FABP,: P55050), the adipocyte isoform FABP4 (A-FABP; P04117) and the epidermal isoform FABP5 (E-FABP; Q05816), (2) enzymes of malonyl-CoA metabolism, such as acetyl-CoA carboxylase isoforms ACC1 (Q5SWU9) and ACC2 (E9Q4Z2) and fatty acid synthase (P19096), (3) acetyl-CoA transfer proteins, including the mitochondrial ACAT1 (Q8QZT1) and cytosolic ACAT2 (Q8CAY6) isoforms of acetyl-CoA acetyltransferase and liver carnitine O-palmitoyl-transferase CPTA1 (P97742), (4) proteins involved in fatty acid oxidation, including various mitochondrial short, branched, medium, long and very long chain specific acyl-CoA dehydrogenases (P51174, Q8K370, Q8JZN5, P45952, Q07417, P50544, Q9DBL1, Q80XL6) and 3-hydroxyacyl-CoA dehydrogenase (O08756), and (5) enzymes of ketone body metabolism, including the cytoplasmic HMGCS1 isoform (Q8JZK9) and mitochondrial HMGCS2 isoform (P54869) of hydroxyl-methylglutaryl-CoA synthase.

### Proteomic profiling of liver tissue from the dystrophic *mdx*-*4cv* model of dystrophinopathy

**Fig. 2:**
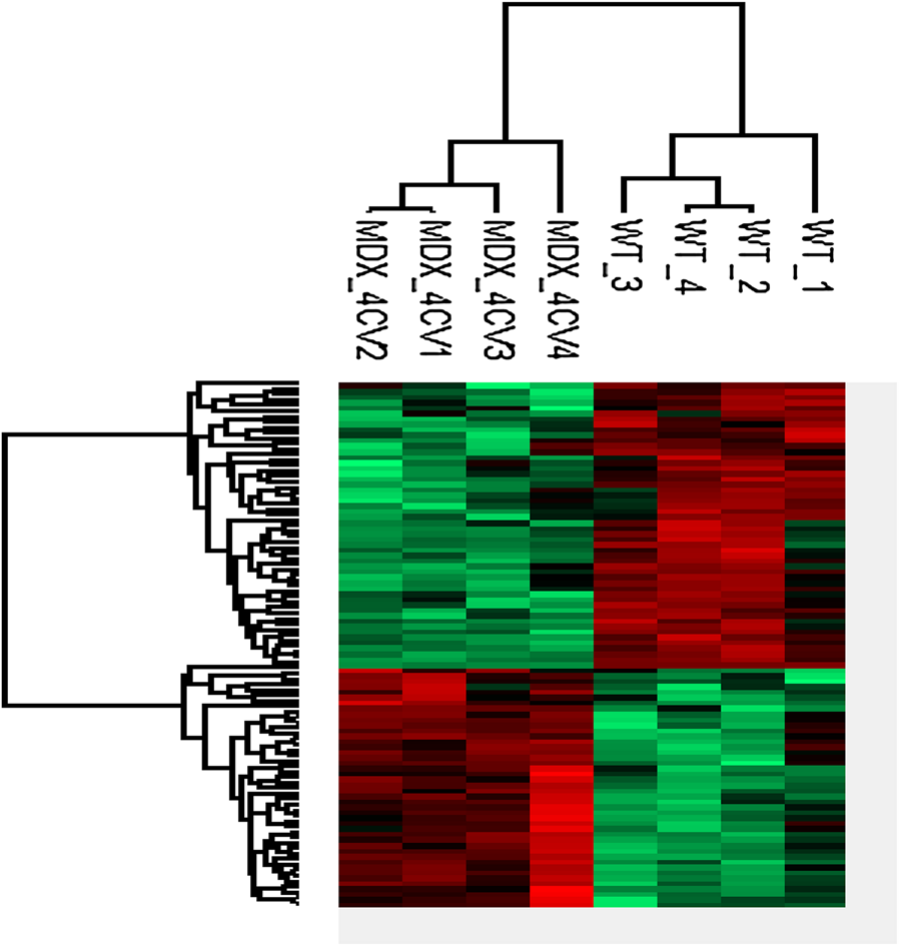
Heat map of differentially expressed proteins in the *mdx*-*4cv* liver. Shown is the clustering of significantly increased versus decreased proteins in liver tissue from the dystrophic *mdx*-*4cv* mouse model of Duchenne muscular dystrophy, as compared to control tissues. For the identification of proteome-wide changes in the liver, protein extracts from whole tissue preparations of 6-month-old *mdx*-*4cv mice* (n = 4; MDX-4CV 1–4) versus age-matched wild type mice (n = 4; WT 1–4) were analysed by mass spectrometry-based proteomics

The comparative mass spectrometric analysis of wild type versus *mdx*-*4cv* liver extracts identified a total of 96 proteins with a significant change in abundance. Of these liver-associated protein species, 44 proteins were found to be increased (Table 1) and 52 proteins were shown to be decreased (Table 2) in their concentration. The protein confidence score listed in the tables is the combined score of the peptides identified by SEQUEST. Therefore, if a protein is identified by 3 peptides, the Progenesis program adds up the three XCorr scores defined by SEQUEST and uses these values as a confidence score for the protein result. The listings of proteomic hits included 37 liver proteins with an altered expression level above 2-fold. A heat map is shown in Fig. 2 that outlines the clustering of significantly increased versus decreased proteins in *mdx*-*4cv* liver tissue, as compared to control tissues. The most drastically increased proteins, which did not exhibit differential effects in a closely related isoform, were identified as the FABP5 isoform of fatty acid-binding protein, the calcium binding protein calumenin, triokinase, aldolase and epiplakin, as well as the light and heavy chains of the iron binding protein ferritin (Table 1). In contrast, the most reduced proteins, which did not exhibit differential effects in a closely related isoform, were shown to be the glycolytic enzyme phosphoglycerate kinase PGK1, apolipoprotein A-II, galectin-1, fatty aldehyde dehydrogenase, cysteine sulfinic acid decarboxylase, acyl-coenzyme A thioesterase 1 and mitochondrial ornithine aminotransferase, as well as the modulators of lipid metabolism perilipin-2 and perilipin-3 (Table 2). A few isoforms of related protein species showed differential changes in the *mdx*-*4cv* liver, including cytochrome P450-2C29/P450-2C54 versus P450-3A11, Ig alpha chain C region versus gamma-2B chain C region, and glutathione S-transferase P1 versus A1.

**Table 1:** Proteomic identification of liver proteins with an increased abundance in the *mdx*-*4cv* model of dystrophinopathy

Accession	Gene name	Description	Unique peptides	Confidence score	Anova (*p*)	Max fold change
Q05816	Fabp5	Fatty acid-binding protein, epidermal	3	10.5609	0.00875	19.78
Q64458	Cyp2c29	Cytochrome P450 2C29	2	6.5311	0.01688	3.75
P01878	N/A	Ig alpha chain C region	2	9.0393	0.00267	3.27
P29391	Ftl1	Ferritin light chain 1	8	23.6657	0.02194	3.00
O35887	Calu	Calumenin	2	7.26614	0.02929	2.99
P09528	Fth1	Ferritin heavy chain	4	25.8182	0.00734	2.81
Q8VC30	Tkfc	Triokinase/FMN cyclase	2	6.2330	0.02067	2.66
P05063	Aldoc	Fructose-bisphosphate aldolase C	3	7.7044	0.00265	2.40
Q8K182	C8a	Complement component C8 alpha chain	3	12.0831	0.01038	2.24
Q8R0W0	Eppk1	Epiplakin	5	20.9671	0.00183	2.18
Q61490	Alcam	CD166 antigen	2	5.2107	0.00250	2.17
Q01279	Egfr	Epidermal growth factor receptor	5	18.7714	0.01288	2.15
Q6XVG2	Cyp2c54	Cytochrome P450 2C54	3	11.5034	0.00478	2.11
P28666	Mug2	Murinoglobulin-2	3	8.3401	0.01112	2.02
O08807	Prdx4	Peroxiredoxin-4	5	22.4464	0.00022	1.95
Q9WVJ3	Cpq	Carboxypeptidase Q	2	5.6121	0.02628	1.92
Q8CFX1	H6pd	GDH/6PGL endoplasmic bifunctional protein	4	12.9820	0.00227	1.90
Q6P5E4	Uggt1	UDP-glucose:glycolprotein glucosyltransferase 1	2	7.1692	0.00337	1.89
Q3UPL0	Sec31a	Protein transport protein Sec31A	2	7.6000	0.00183	1.88
Q60866	Pter	Phosphotriesterase-related protein	4	14.1754	0.01619	1.87
P17563	Selenbp1	Selenium-binding protein 1	14	61.3323	0.00128	1.82
P19157	Gstp1	Glutathione S-transferase P 1	5	19.3699	0.00183	1.79
P55302	Lrpap1	Alpha-2-macroglobulin receptor-associated protein	3	9.5635	0.01538	1.76
O08600	Endog	Endonuclease G, mitochondrial	3	9.6149	0.00536	1.74
Q61694	Hsd3b5	3 beta-hydroxysteroid dehydrogenase type 5	2	7.7579	0.01446	1.73
Q922R8	Pdia6	Protein disulfide-isomerase A6	5	16.2039	0.00687	1.71
Q8BTY1	Kyat1	Kynurenine–oxoglutarate transaminase 1	5	20.5273	0.00447	1.71
Q8VCU1	Ces3b	Carboxylesterase 3B	5	15.2176	0.00868	1.71
Q99P27	Pla2g12b	Group XIIB secretory phospholipase A2-like protein	2	8.1664	0.02943	1.70
P05064	Aldoa	Fructose-bisphosphate aldolase A	7	24.4402	0.00015	1.69
Q9QXS1	Plec	Plectin	4	12.2329	0.00367	1.69
Q921X9	Pdia5	Protein disulfide-isomerase A5	5	19.1847	0.00385	1.66
P05784	Krt18	Keratin, type I cytoskeletal 18	3	10.1094	0.02249	1.65
Q9JKR6	Hyou1	Hypoxia up-regulated protein 1	7	26.4824	0.00022	1.64
Q9CPT4	Mydgf	Myeloid-derived growth factor	2	6.4866	0.01417	1.62
P52840	Sult1a1	Sulfotransferase 1A1	3	11.3351	0.00914	1.62
P20029	Hspa5	78 kDa glucose-regulated protein	9	32.5662	0.00432	1.61
Q9QZ85	Iigp1	Interferon-inducible GTPase 1	3	9.9380	0.00171	1.60
Q8VDD5	Myh9	Myosin-9	18	69.8377	0.00808	1.58
Q8BL66	Eea1	Early endosome antigen 1	4	15.6173	0.00058	1.56
P20152	Vim	Vimentin	4	11.0748	0.00435	1.55
P16675	Ctsa	Lysosomal protective protein	2	10.4083	0.00248	1.54
P24369	Ppib	Peptidyl-prolyl cis–trans isomerase B	4	13.1642	0.02870	1.53
Q99PL5	Rrbp1	Ribosome-binding protein 1	19	64.9991	0.00166	1.50

**Table 2:** Proteomic identification of liver proteins with a decreased abundance in the *mdx*-*4cv* model of dystrophinopathy

Accession	Gene name	Description	Unique peptides	Confidence score	Anova (*p*)	Max fold change
P09411	Pgk1	Phosphoglycerate kinase 1	4	13.9587	2.53E−09	14.97
Q64459	Cyp3a11	Cytochrome P450 3A11	5	17.1715	0.00056	9.53
P09813	Apoa2	Apolipoprotein A-II	2	9.5295	0.00130	6.98
P16045	Lgals1	Galectin-1	2	4.2800	0.00138	6.51
P47740	Aldh3a2	Fatty aldehyde dehydrogenase	7	24.1473	0.00055	6.02
P43883	Plin2	Perilipin-2	4	10.1732	0.02351	5.71
Q9DBE0	Csad	Cysteine sulfinic acid decarboxylase	13	41.5240	0.00220	5.63
P13745	Gsta1	Glutathione S-transferase A1	2	4.6699	0.02167	4.22
P01867	Igh-3	Ig gamma-2B chain C region	3	8.7297	0.01044	3.48
O55137	Acot1	Acyl-coenzyme A thioesterase 1	4	17.5564	0.00199	3.39
P29758	Oat	Ornithine aminotransferase, mitochondrial	22	74.8421	0.00012	2.79
Q9DBG5	Plin3	Perilipin-3	2	5.2382	0.01285	2.63
Q91VS7	Mgst1	Microsomal glutathione S-transferase 1	4	10.5855	0.00520	2.57
P06801	Me1	NADP-dependent malic enzyme	4	11.4988	0.01467	2.44
Q8VCH0	Acaa1b	3-ketoacyl-CoA thiolase B, peroxisomal	10	39.1412	0.00755	2.37
P22599	Serpina1b	Alpha-1-antitrypsin 1-2	3	7.0562	0.00500	2.34
Q9QZX7	Srr	Serine racemase	2	6.2347	0.02590	2.24
O54754	Aox1	Aldehyde oxidase 1	2	6.4066	0.00869	2.21
Q8BGT5	Gpt2	Alanine aminotransferase 2	8	26.9779	5.36E−05	2.17
Q8R086	Suox	Sulfite oxidase, mitochondrial	13	55.1200	0.00039	2.12
P16015	Ca3	Carbonic anhydrase 3	6	20.1812	0.00752	2.11
Q9D379	Ephx1	Epoxide hydrolase 1	3	8.8219	0.00583	2.11
P07759	Serpina3 k	Serine protease inhibitor A3 K	2	8.0261	0.00156	2.04
Q8VBT2	Sds	l-serine dehydratase/l-threonine deaminase	3	8.3410	0.04215	1.97
Q9QZD8	Slc25a10	Mitochondrial dicarboxylate carrier	6	22.5548	0.00119	1.94
Q91X83	Mat1a	S-adenosyl-methionine synthase isoform 1	7	21.4321	0.01511	1.92
Q9QXX4	Slc25a13	Calcium-binding mitochondrial carrier protein Aralar2	2	8.3671	0.02466	1.87
Q99L20	Gstt3	Glutathione S-transferase theta-3	6	23.9548	0.00840	1.86
Q8CAQ8-5	Immt	Isoform 5 of MIC complex subunit Mic60	4	11.5137	0.00397	1.86
Q922B1	Macrod1	O-acetyl-ADP-ribose deacetylase MACROD1	2	6.7757	0.00065	1.86
Q9D7I5	Lhpp	Phospholysine phosphohistidine inorganic pyrophosphate phosphatase	3	8.5881	0.00382	1.85
Q921H8	Acaa1a	3-ketoacyl-CoA thiolase A, peroxisomal	2	8.07142	0.00063	1.84
Q64176	Ces1e	Carboxylesterase 1E	3	12.0204	0.01780	1.79
P61922	Abat	4-aminobutyrate aminotransferase, mitochondrial	7	24.4437	0.00016	1.75
O35490	Bhmt	Betaine–homocysteine S-methyltransferase 1	4	13.8152	0.03353	1.73
Q8BUV3	Gphn	Gephyrin	2	5.9008	0.01958	1.69
P02088	Hbb-b1	Hemoglobin subunit beta-1	2	7.9579	0.03523	1.67
P13707	Gpd1	Glycerol-3-phosphate dehydrogenase, cytoplasmic	9	30.8543	0.01008	1.63
Q91WG0	Ces2c	Acylcarnitine hydrolase	4	12.3891	0.00150	1.63
P15327	Bpgm	Bisphospho-glycerate mutase	3	10.8651	0.01946	1.61
P15626	Gstm2	Glutathione S-transferase Mu 2	3	8.9072	0.02081	1.60
Q9D0F9	Pgm1	Phosphogluco-mutase-1	2	7.4358	0.03949	1.60
Q99K51	Pls3	Plastin-3	3	15.7664	0.00796	1.58
Q9QXF8	Gnmt	Glycine N-methyltransferase	4	13.7679	0.00466	1.57
P41216	Acsl1	Long-chain-fatty-acid–CoA ligase 1	13	41.6748	0.00427	1.57
Q9DCM0	Ethe1	Persulfide dioxygenase ETHE1, mitochondrial	3	7.8214	7.48E−05	1.56
O35678	Mgll	Monoglyceride lipase	3	9.8837	0.00490	1.55
Q9WUZ9	Entpd5	Ectonucleoside triphosphate diphospho-hydrolase 5	2	6.6691	0.00680	1.55
Q9WVL0	Gstz1	Maleylacetoacetate isomerase	2	6.1377	0.00435	1.54
Q8CC88	Vwa8	von Willebrand factor A domain-containing protein 8	11	35.6611	0.00040	1.54
P34928	Apoc1	Apolipoprotein C-I	2	7.9163	0.00828	1.54
O08756	Hsd17b10	3-hydroxyacyl-CoA dehydrogenase 2	3	10.6184	0.00691	1.53

### Bioinformatics analysis of global protein changes in the *mdx*-*4cv* liver

**Fig. 3:**
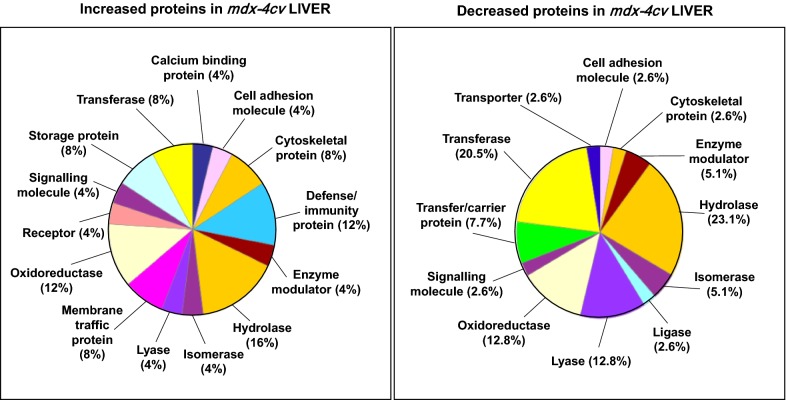
Summary of changed protein classes in liver tissue extracts from the *mdx*-*4cv* mouse model of dystrophinopathy. In order to identify the clustering of protein classes based on the mass spectrometric analysis of wild type versus *mdx*-*4cv* liver (Tables 1, 2), the bioinformatics software programme PANTHER [41] was used

**Fig. 4:**
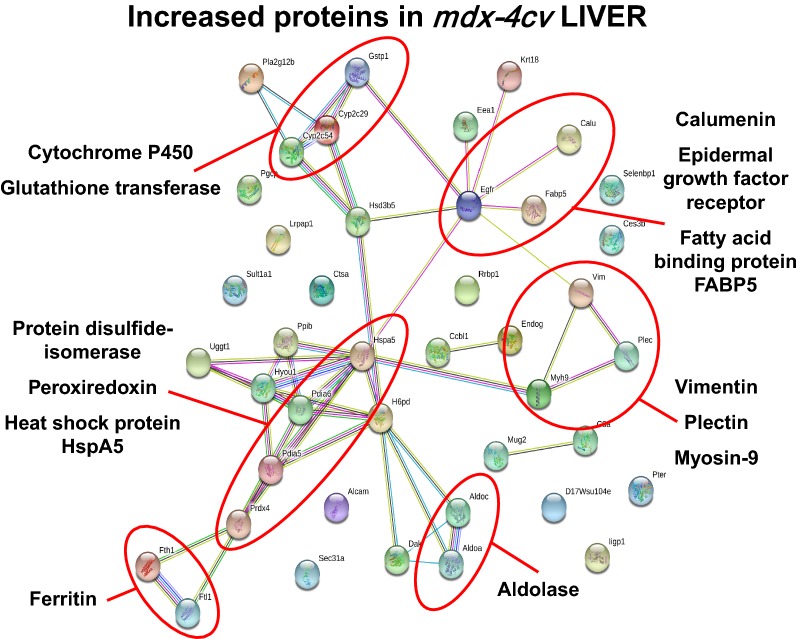
Overview of potential protein interaction patterns between increased proteins in liver tissue extracts from the *mdx*-*4cv* mouse model of dystrophinopathy. In order to identify protein interactions, based on both direct physical and indirect functional protein associations, of the mass spectrometrically identified proteins from wild type versus *mdx*-*4cv* liver (Table 1), the bioinformatics software programme STRING [42] was used

**Fig. 5:**
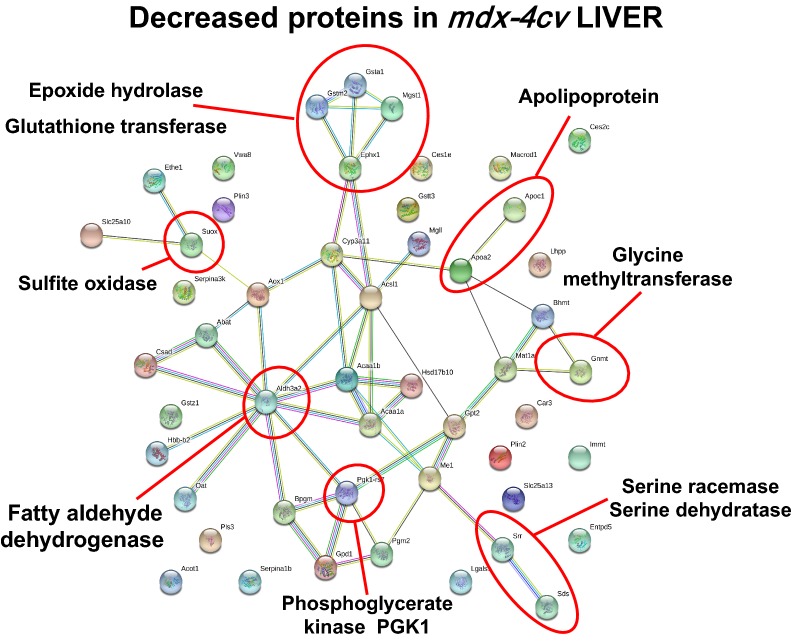
Overview of potential protein interaction patterns between decreased proteins in liver tissue extracts from the *mdx*-*4cv* mouse model of dystrophinopathy. In order to identify protein interactions, based on both direct physical and indirect functional protein associations, of the mass spectrometrically identified proteins from wild type versus *mdx*-*4cv* liver (Table 2), the bioinformatics software programme STRING [42] was used

The proteomic data sets from the comparative analysis of liver tissue from wild type versus *mdx*-*4cv* mice were analysed by standard bioinformatics software programs, i.e. the PANTHER database of protein families and the STRING database of protein interactions that include both direct physical and indirect functional protein associations, in order to group the identified liver-associated proteins based on their protein class and to evaluate potential protein interaction patterns. As outlined in the PANTHER analysis graph of Fig. 3, the proteomic survey of *mdx*-*4cv* liver extracts revealed considerable alterations in a variety of enzymes belonging to the classes of hydrolases, oxidoreductases and transferases, as well as proteins involved in membrane trafficking, cell adhesion, cytoskeletal maintenance, cellular defence and immunity (Tables 1, 2). Potential interaction maps of altered proteins were established by STRING analyses, which indicate a few protein hubs of potential importance for explaining the perturbed protein expression patterns in the *mdx*-*4cv* liver. Protein clustering of increased proteins appears to occur at the level of certain isoforms of cytochrome P450 and glutathione transferase, protein disulfide-isomerase, peroxiredoxin and heat shock protein HspA5, ferritin light and heavy chains, aldolase isoforms, vimentin, plectin and myosin-9, and the epidermal growth factor receptor, calumenin and fatty acid binding protein FABP5 (Fig. 4). This is indicative of increased biotransformation, molecular chaperoning and cellular signalling. Protein hubs with components of decreased abundance centre around epoxide hydrolase and glutathione transferase isoforms, sulphide oxidase, fatty aldehyde dehydrogenase, serine racemase and dehydratase, glycine methyltransferase, apolipoproteins, and phosphoglycerate kinase PGK1 (Fig. 5).

### Drastic increase of fatty acid binding protein FABP5 in the *mdx-4cv* liver

**Fig. 6:**
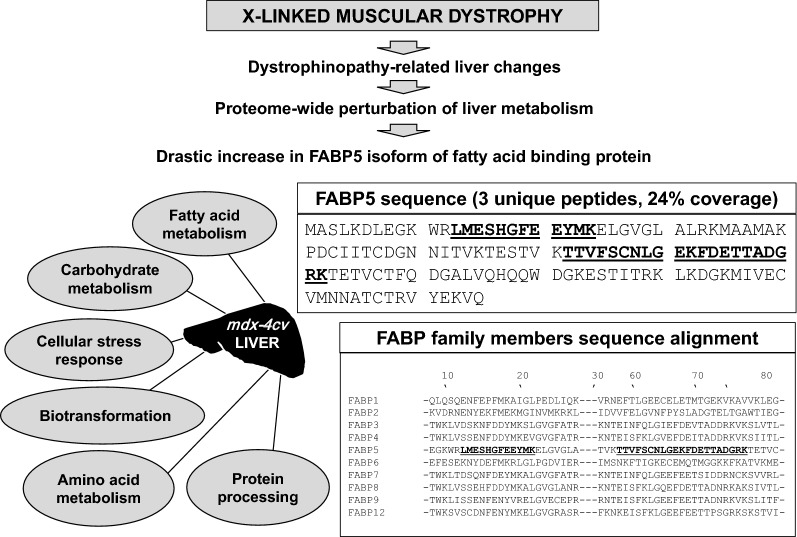
Overview of the proteomic identification of the fatty acid binding protein FABP5 in liver tissue extracts from the *mdx*-*4cv* mouse model of dystrophinopathy. Shown is the potential correlation between primary skeletal muscle abnormalities in X-linked muscular dystrophy and secondary changes in liver metabolism, as well as an overview of affected biochemical pathways in the *mdx*-*4cv* liver as determined by LS-MS/MS analysis. The mass spectrometric fingerprint of the most increased protein species in the *mdx*-*4cv liver*, FABP5, is shown and a comparative sequence alignment with other members of the FABP family to illustrate the uniqueness of the identifying peptide sequence of FABP5

A major finding of this study was the drastic increase in FABP5, which was identified by 3 unique peptides. The sequence alignment of the protein family of FABPs in *Mus musculus* is shown in Fig. 6. FABP isoforms 1–9 and 12 represent the proteins located in the liver (L-FABP), intestine (I-FABP), heart (H-FABP), adipocytes (A-FABP), epidermal tissue (E-FABP), ileum (Il-FABP), brain (B-FABP), myelin (M-FABP) and testis (T-FABP), respectively. The mass spectrometric identification shows that the identifying peptides are unique to isoform FABP5 (Fig. 6). The epidermal isoform FABP5, also known as keratinocyte-type FABP or psoriasis-associated-FABP, has been found in a great variety of organs, tissues and cell types including skin, liver, tongue, mammary glands, brain, stomach, intestine, kidney, lung, heart, skeletal muscle, testis, retina, lens, spleen, placenta, adipocytes, macrophages and dendritic cells [45]. This agrees with the proteomic identification of FABP5 in wild type and *mdx*-*4cv* liver.

### Immunoblot analysis of fatty acid binding protein FABP5 in the *mdx*-*4cv* liver

**Fig. 7:**
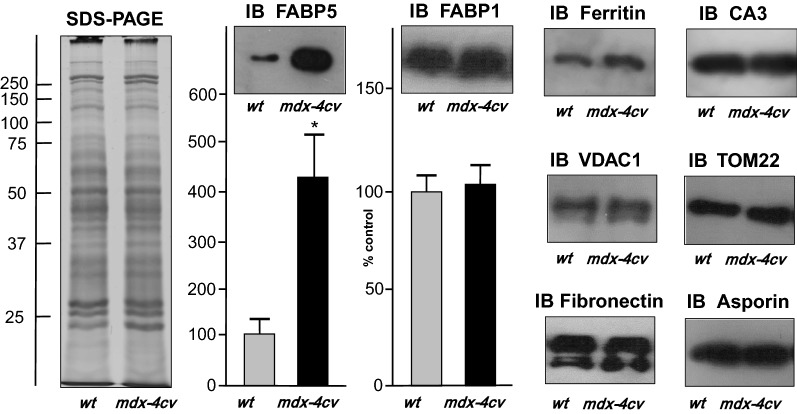
Comparative immunoblot analysis of normal wild type versus *mdx*-*4cv* liver extracts. Shown is a representative silver-stained SDS-PAGE gel and corresponding immunoblots with expanded views of labelling with antibodies to the fatty acid binding protein isoforms FABP1 and FABP5, as well as ferritin light chain, carbonic anhydrase isoform CA3, the voltage-dependent anion channel VDAC-1, the mitochondrial outer membrane protein translocation pore subunit TOM22, fibronectin and asporin. Lanes 1 and 2 represent total extracts from control wild type (*wt*) liver and *mdx*-*4cv* liver, respectively. Molecular mass standards (in kDa) are indicated at the left of the gel image. Graphical representations of the immuno-decoration levels of FABP1 and FABP5 are shown (Y-axis: % control): Student’s *t* test, unpaired; n = 4; *p < 0.05

To independently verify the status of the most significantly increased liver-associated protein species, as determined by comparative proteomics, immunoblot analysis and immunofluorescence microscopy was carried out with a focus on the FABP5 isoform of fatty acid binding protein. The silver-stained gel in Fig. 7 showed no major changes in the overall protein banding pattern between wild type and *mdx*-*4cv* liver extracts. However, the immunoblotting of FABP isoforms using specific antibodies to FABP1 versus FABP5 confirmed the significant increase of the FABP-5 (E-FABP) isoform in the *mdx*-*4cv* liver, but showed no major changes in the abundance of the FABP-1 (L-FABP) isoform (Fig. 7). The proteomic identification of increased levels of liver-associated ferritin light chain agreed with a tendency of an increased concentration of the iron-binding protein as determined by immunoblotting, but these findings were not shown to be statistically significant. In addition, immunoblotting was used to demonstrate the comparable concentration of a variety of liver-associated marker proteins, including the CA3 isoform of carbonic anhydrase, the voltage-dependent anion channel VDAC-1; the mitochondrial outer membrane protein translocation pore subunit TOM22, fibronectin and asporin (Fig. 7).

### Immunofluorescence microscopical analysis of *mdx*-*4cv* skeletal muscle and liver

**Fig. 8:**
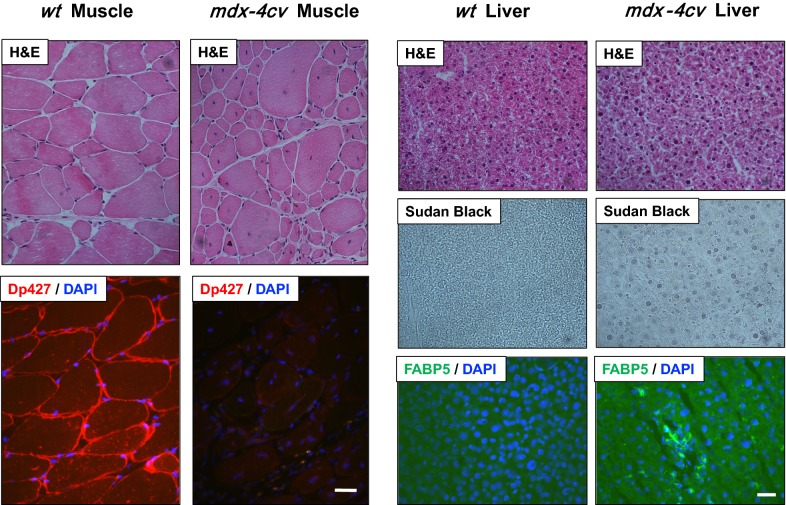
Histological and immunofluorescence microscopical analysis of wild type versus *mdx*-*4cv* muscle and liver. Shown is the select labelling of transverse cryosections from normal wild type (*wt*) versus *mdx*-*4cv gastrocnemius* muscle and liver with haematoxylin/eosin (H&E), as well as antibodies to dystrophin isoform Dp427 and the fatty acid binding protein isoform FABP5. Liver sections were also labelled with Sudan Black dye. Bars equal 50 μm

To establish the mutant status of the genetic mouse model used in this comparative proteomic study of the *mdx*-*4cv* liver, histological changes in the skeletal musculature were assessed by standard haematoxylin/eosin staining and the deficiency in dystrophin isoform Dp427-M was confirmed by immunofluorescence microscopy [27]. Figure 8 illustrates that dystrophin deficiency is associated with a greater variety in fibre diameter in the *mdx*-*4cv gastrocnemius* muscle. The drastic increase in central nucleation in *mdx*-*4cv* muscle fibres was visualized by haematoxylin/eosin staining. Sudan Black staining of the *mdx*-*4cv* liver showed specific changes in the cellular composition of hepatocytes, indicated by increased levels of blue-black staining patterns that are representative of fat deposition in the liver from dystrophic mice (Fig. 8). The immunofluorescence microscopical comparison of FABP5 in wild type versus *mdx*-*4cv* liver tissue revealed increased levels in the dystrophic model. Although the fluorescent labelling of FABP5 is relatively weak, the elevated staining pattern in the *mdx*-*4cv* liver agreed with the findings from the proteomic survey and the immunoblot analysis of this protein isoform.

## Discussion

As the largest multi-functional internal organ in the body, the liver has a very broad variety of biochemical functions, including digestion, nutrient storage, the integration of key metabolic pathways, the essential detoxification of xenobiotic substances, protein synthesis and immunity [46]. The transcriptome and proteome of human liver tissue has been extensively categorised by large-scale profiling studies [47–50] and detailed bioinformatics databases have been established that specifically focus on molecular hepatology in health and disease [51, 52]. Building on these data banks of liver protein expression, including findings from detailed cataloguing studies of the mouse liver proteome [53–55], we have used here a comparative proteomic approach to study potential changes in liver proteins due to X-linked muscular dystrophy. Drastic changes in the *mdx*-*4cv* liver were established for protein species involved in fatty acid metabolism, metabolite transportation, carbohydrate metabolism, amino acid metabolism, protein processing, ion handling, biotransformation and the cellular stress response. The finding that of all of the mass spectrometrically detected proteins only relatively few were identified as being differentially expressed is most likely due to the fact that the *mdx*-*4cv* liver is only indirectly affected in muscular dystrophy. In contrast to the drastic and primary effect of the loss of the full-length muscle isoform of dystrophin in contractile tissues, non-muscle cells and tissues that do not express the full-length dystrophin isoform might thus only exhibit relatively few proteome-wide changes. Instead secondary changes in metabolism might represent the main response of the liver to primary dystrophin deficiency-induced alterations in skeletal muscle, cardiac tissue, the central nervous system and the circulatory system.

One of the most intriguing findings of this study is the striking increase in fatty acid binding protein FABP5, which is indicative of specific changes in the binding of long-chain fatty acids and other lipophilic ligands [56], as well as alterations in associated cellular signalling events [57]. Interestingly, the liver isoform FABP1 was shown not to be majorly affected in liver tissue in dystrophinopathy. Of note, a variety of FABP isoforms, including FABP1, FABP3 and FABP5, were identified by proteomics in serum from the *mdx*-*4cv* mouse [32] and Duchenne patients [58–60]. Since the well-characterized FABP isoforms 1–9 and 12 have a broad expression pattern in many tissues and organs throughout the body [45], it is not possible to properly correlate serum changes in individual FABP isoforms to specific pathophysiological shedding events in particular organs. FABPs belong to the class of intracellular lipid-binding proteins that are primarily involved in the reversible binding of hydrophobic ligands and their subcellular trafficking through major organellar systems [45]. In addition, individual FABPs have also myokine and adipokine functions that regulate signalling events in relation to body-wide metabolic control. The drastic increase of FABP5 in the *mdx*-*4cv* liver would suggest this protein as a potential biomarker candidate of liver dysfunction in X-linked muscular dystrophy. However, changes in FABPs were previously shown to occur in many common disorders. Dysregulation was established for FABP3 in cardiovascular disorders [56, 61], FABP1, FABP2, FABP4 and FABP5 in non-alcoholic fatty liver disease [56, 62] and FABP5 and FABP7 in cancer [56, 63, 64]. Although this excludes the dystrophinopathy-associated change in liver FABP5 as a robust and highly specific biomarker of X-linked muscular dystrophy, altered FABP5 levels may nevertheless be useful as a general pathophysiological marker in conjunction with other disease indicators.

In general, enzymes involved in fatty acid metabolism were differentially affected in the *mdx*-*4cv* liver, including decreases in apolipoprotein A-II, fatty aldehyde dehydrogenase and acyl-coenzyme A thioesterase 1. Apolipoproteins are essential mediators of lipid binding and transportation through the lymphatic and circulatory systems, but also function as receptor ligands and enzyme cofactors. A key component of the detoxification pathway of saturated and unsaturated aldehydes, which arise from lipid peroxidation, is the fatty aldehyde dehydrogenase that converts long-chain aliphatic aldehydes to fatty acids. The regulation of intracellular levels of acyl-CoA and free fatty acids is related to the activity levels of acyl-CoA thioesterases, which catalyses the hydrolysis of acyl-CoA molecules to coenzyme A and free fatty acids [65, 66]. In addition, the essential modulators of lipid metabolism, perilipin-2 and perilipin-3, were shown to be reduced in the *mdx*-*4cv* liver. During lipogenesis in the liver, excess energy is stored as triacylglycerol units in the form of lipid droplets, and this stored energy is released again by hydrolysis in lipolytic pathways [67]. The family of cytosolic lipid droplet coat proteins, named perilipin PLIN1 to 5, coordinate this process of lipid storage, reactivation and utilization. Pathophysiological imbalances of lipid droplet homeostasis may trigger lipocytotoxicity [68]. Thus, the changed perilipin levels identified in this proteomic study suggest that the physiological regulation of lipid accumulation and release of triacylglycerol appears to be altered in the liver in muscular dystrophy. This may play a role in the overall perturbation of lipid metabolism in dystrophinopathy.

The proteomic profile of the *mdx*-*4cv* liver showed differential changes in the concentration of enzymes involved in cellular carbohydrate metabolism, including increases in triokinase, which catalyses the phosphorylation of both glyceraldehyde and dihydroxyacetone [69]. A disturbed regulation of carbohydrate metabolism may be the underlying cause of decreased liver glycogen levels [19] and a mild glucose intolerance in dystrophinopathy [20, 21]. Opposite concentration changes were established for two key enzymes of glycolysis, i.e. increases in fructose-bisphosphate aldolase and decreases in phosphoglycerate kinase. Aldolase mediates the reversible reaction that breaks down fructose-1,6-biphosphate into dihydroxyacetone phosphate and glyceraldehyde-3-phosphate in the glycolytic pathway. Phosphoglycerate kinase catalysis a crucial ATP-generating transfer reaction in glycolysis by forming 3-phosphoglycerate from ADP and 1,3-bisphosphoglycerate. During gluconeogenesis, phosphoglycerate kinase catalyses the reverse reaction, making it a key regulator of glucose metabolism [70]. Differential changes in glycolytic enzymes were also observed in dystrophic skeletal muscle and cardiac tissue [29, 33, 36, 71, 72]. These findings suggest considerable secondary and body-wide changes in carbohydrate metabolism due to dystrophin deficiency. However, besides their metabolic functions, glycolytic enzymes exhibit a multifaceted role in transcriptional regulation, stimulation of cell motility and the regulation of cell death [73]. These non-glycolytic functions appear to link metabolism to epigenetic and transcription programs [74] and might also be affected in dystrophinopathy.

Besides alterations in key metabolic processes, mass spectrometry identified significant increases in two essential ion-binding proteins, e.g. calumenin and ferritin. Their elevated levels in the *mdx*-*4cv* liver might present compensatory mechanisms to counter-act abnormal calcium handling and iron toxicity. Calumenin is a relatively low-affinity Ca^2+^-binding protein, which plays a biochemical role in protein folding and sorting mechanisms in the endoplasmic reticulum. In the secretory pathway of the endoplasmic reticulum, calumenin regulates the activity of the SERCA2a isoform of the Ca^2+^-pumping ATPase and the ryanodine receptor Ca^2+^-release channel [75]. Hence, up-regulation of calumenin might counter-act disturbed calcium homeostasis in the *mdx*-*4cv* liver. In analogy, the increased concentration of the light and heavy chains of liver ferritin might act as a cytosolic iron-buffering system in the *mdx*-*4cv* liver that prevents a detrimental iron imbalance. Both, iron deficiency or iron overload may trigger abnormal cellular functions [76] and therefore has to be properly balanced by abundant intracellular iron-binding proteins [77]. The ferritin type of iron-binding proteins sequester and release iron in a highly regulated way and serve in most tissues a dual physiological function, which provides both a pathway for efficient iron detoxification and storage of a cellular iron reserve [78]. Besides the liver, ferritin light and heavy chains were previously also shown to be drastically increased in dystrophin-deficient heart tissue [72] and skeletal muscle [29, 79, 80]. In addition, urinary ferritin levels are greatly increased in young Duchenne patients and are suggested to be functionally linked to the renal management of myoglobin iron that derived from the disintegration of dystrophic muscles [81]. These findings suggest that elevated levels of ferritin in the dystrophic phenotype act as a physiological buffer against a potential iron overload.

Besides the above discussed alterations in mostly metabolic proteins, including abundant enzymes, transporters and binding proteins, less pronounced changes were identified in various markers of cytoskeletal maintenance, biotransformation, anti-oxidative mechanisms, protease activity, regulation of cellular growth and differentiation, and the cellular stress response. Established markers of this wide range of processes in the liver are epiplakin, cytochrome P450 isoforms, glutathione transferases, peroxiredoxin, carboxypeptidase, alpha-1-antitrypsin, epidermal growth factor receptor, galectin, protein disulfide isomerase and molecular chaperones. These complex and proteome-wide changes in the *mdx*-*4cv* liver suggest general perturbations of hepatic function in X-linked muscular dystrophy. Since increasing dietary taurine was shown to increase contractile strength in dystrophinopathy [17], it will be interesting to evaluate in future studies whether elevated taurine levels trigger an anti-inflammatory, anti-oxidant and/or cyto-protective response [18] and possibly improve transport and metabolism of this amino acid in the liver from dystrophic organisms [16].

## Conclusions

Previous studies on liver abnormalities in Duchenne muscular dystrophy have established that liver atrophy occurs concurrently with skeletal muscle wasting and that this secondary pathology may be linked to heart failure in older patients suffering from dystrophinopathy. In addition, an increased susceptibility to drug-induced hepatotoxicity is associated with X-linked muscular dystrophy. The comparative proteomic study of the dystrophic *mdx*-*4cv* mouse model of dystrophinopathy outlined in this report confirms liver abnormalities and identified expression changes in a variety of liver proteins due to dystrophin deficiency. The systematic mass spectrometric survey of the *mdx*-*4cv* liver suggests that liver metabolism is perturbed and increased levels of the fatty acid binding protein FABP5 appear to be a major pathobiochemical feature of the dystrophic *mdx*-*4cv* phenotype. A variety of changes in other metabolic enzymes, binding proteins and transporters may present adaptations or compensatory mechanisms to prevent cellular complications due to lipocytotoxicity or iron overload in an organism that lacks the membrane cytoskeletal protein dystrophin.
